# c‐Abl kinase regulates cell proliferation and ionizing radiation‐induced G2/M arrest via phosphorylation of FHL2

**DOI:** 10.1002/2211-5463.13177

**Published:** 2021-05-19

**Authors:** Guang‐Fei Wang, Xiayang Niu, Hainan Liu, Qincai Dong, Yebao Yao, Di Wang, Xuan Liu, Cheng Cao

**Affiliations:** ^1^ Beijing Institute of Biotechnology China; ^2^ Anhui University Hefei China

**Keywords:** c‐Abl, cell proliferation, FHL2, G2/M arrest, phosphorylation

## Abstract

Nonreceptor tyrosine kinase c‐Abl participates in several cellular processes by phosphorylating transcription factors or cofactors. c‐Abl binds and phosphorylates four‐and‐a‐half‐LIM‐only protein 2 (FHL2), but the identity of the phosphorylation sites and their contribution to cell cycle regulation is unclear. In this study, we demonstrate that c‐Abl highly phosphorylates FHL2 at Y97, Y176, Y217, and Y236 through mass spectrometry and tyrosine‐to‐phenylalanine (Y → F) mutant analysis. Proliferation was inhibited in cells expressing wild‐type (WT) FHL2 but not cells expressing the phosphorylation‐defective mutant FHL2(4YF). Moreover, FHL2 contributed to cell cycle arrest at G2/M induced by ionizing radiation (IR). FHL2 WT but not FHL2(4YF) rescued FHL2 function in FHL2‐depleted cells by causing IR‐induced G2/M arrest. These results demonstrate that c‐Abl regulates cell cycle progression by phosphorylating FHL2.

## Graphical abstract

c‐Abl kinase binds and phosphorylates FHL2, but the identity of the phosphorylation sites and their contribution to cell cycle regulation is unclear. We demonstrate that c‐Abl highly phosphorylates FHL2 at Y97, Y176, Y217, and Y236. c‐Abl‐mediated FHL2 phosphorylation can inhibit cell proliferation under physiological conditions and cell cycle arrest at G2/M induced by ionizing radiation (IR).

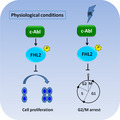

**Abbreviations**
CBB: Coomassie Brilliant Blue
DMEM: Dulbecco’s modified Eagle’s medium
FHL: four‐and‐a‐half‐LIM
IR: ionizing radiation
LC–MS/MS: liquid chromatography tandem–mass spectrometry
WT: wild‐type

The nonreceptor tyrosine kinase c‐Abl, initially known as a proto‐oncogene, is a homolog of the transforming element of the Abelson murine leukemia virus [1]. c‐Abl is a multifunctional protein involved in many cellular processes, such as the regulation of cell proliferation, cell cycle arrest, DNA damage responses, actin dynamics, differentiation, adhesion, migration, and apoptosis [2, 3, 4, 5, 6, 7, 8, 9]. c‐Abl mediates specific gene transcription by binding and phosphorylating their transcription factors or cofactors, such as NF‐κB, p53, p73, C/EBPβ, and Runx1 [10, 11, 12, 13, 14].

FHL2, known as an adaptor protein, belongs to the four‐and‐a‐half‐LIM (FHL) family and is a transcriptional cofactor [15, 16]. It has a higher affinity to multiple functional classes of transcription factors and other cofactors through its LIM domains but does not bind DNA directly. FHL2 regulates the transcription of multiple genes; thus, it is involved in diverse functions, including cell proliferation, cell cycle regulation, cell adhesion, apoptosis, cell mobility, and cytoarchitecture [17, 18, 19]. The FHL2 expression level is highly related to cell cycle progression. The G2/M transition was found to be accelerated when FHL2 expression is suppressed in breast cancer MDA‐MB 231 cells, and the induction of the FHL2 target gene, the cell cycle inhibitor p21, was responsible for this event when cells were treated with the DNA damaging agent doxorubicin [20].

Our previous study found that c‐Abl interacts with and phosphorylates FHL2, but the function of this event remains unclear [21]. In this study, we evaluated c‐Abl‐mediated FHL2 phosphorylation by liquid chromatography tandem–mass spectrometry (LC–MS/MS) and found that defective FHL2 phosphorylation accelerated cell proliferation under physiological conditions and cell cycle release from G2/M arrest induced by ionizing radiation (IR).

## Materials and methods

### Cell culture

Human breast cancer cells (MCF‐7) and embryonic kidney cells (HEK293T) were maintained in Dulbecco's modified Eagle’s medium (DMEM, GIBCO, Grand Island, NY, USA) supplemented with 10% fetal bovine serum (FBS, GIBCO, Grand Island, NY, USA), penicillin (100 U·mL^−1^), and streptomycin (100 μg·mL^−1^). Cells were incubated at 37°C with 5% CO_2_.

#### Plasmids and transfection

Myc‐tagged c‐Abl or Flag‐tagged FHL2 was expressed by cloning the genes into the pcDNA3‐based Flag vector (Invitrogen, Carlsbad, CA, USA) or pCMV‐Myc (Clontech, Mountain View, CA, USA), and their mutants Myc‐c‐Abl (K290R), Flag‐FHL2 (Y93F, Y97F, Y176F, Y217F, Y236F, 4YF), and Cas9‐resistant FHL2 WT/(4YF) silent synonymous mutations were generated by direct mutagenesis (SBS Genetech, Beijing, China). The protein‐expressing plasmids were transfected into HEK293T or MCF‐7 cells using Lipofectamine 2000 or Lipofectamine 3000 according to the manufacturer’s protocol (Life Technologies, Gaithersburg, MD, USA).

#### CRISPR/Cas9‐mediated generation of *fhl2* ^−/−^ cell lines

FHL2 knockout (KO, *fhl2* ^−/−^) cells were generated using the CRISPR/Cas9 system. sgRNA (5′‐UUGCAACGAAUCUCUCUUUG‐3′) was cloned into the pSpCas9(BB)‐2A‐Puro vector (Addgene plasmid ID: 48139), and the constructed plasmid was transfected into MCF‐7 cells using Lipofectamine 3000. FHL2 knockout clones were screened with puromycin and confirmed by genomic sequencing and immunoblotting with an anti‐FHL2 antibody.

#### Immunoprecipitation and immunoblot analysis

Whole cells were lysed using cell lysis buffer (50 mm Tris at pH 7.5, 150 mm NaCl, 1 mm EDTA, 0.1% NP‐40, and protease inhibitor tablets from Roche, Basel, Switzerland). Soluble proteins were subjected to immunoprecipitation with anti‐Flag agarose beads (Sigma‐Aldrich, St. Louis, MO, USA). An aliquot of the total lysate (5%, v/v) was included as the input. Protein samples were separated by SDS/PAGE and transferred onto PVDF membranes. Immunoblot analysis was performed using anti‐Flag (Sigma‐Aldrich, 1:5000), anti‐Myc (Sigma‐Aldrich, 1:5000), anti‐pTyr (Upstate Biotechnology, Lake Placid, NY, USA,1:2000), and anti‐FHL2 (MBL, Tokyo, Japan,1:1000) antibodies.

#### LC–MS/MS analysis

Anti‐Flag antibody immunoprecipitates prepared from HEK293T cells were resolved by SDS/PAGE, and the protein bands were excised. After adequate digestion by trypsin, LC–electrospray ionization–MS/MS‐resolved peptides were analyzed using a Q‐TOF2 system (MicroMass, Manchester, UK), and the data were compared against SWISSPROT using the Mascot search.

#### EdU incorporation assay and flow cytometry analysis

MCF‐7 cells were plated in 60‐mm dishes at a density of approximately 40% and were cultured overnight before additional treatment. For EdU incorporation, the cells were incubated with EdU reagent at a final concentration of 10 μm for 12 h. The cells were then digested with trypsin, washed with PBS and resuspended in 3% FBS, fixed in 5 mL 70% ethanol, and incubated at −20°C for at least 30 min. The cells were stained with Alexa Fluor® 594 using the Click‐iT® EdU assay kit (Invitrogen) and then were subjected to flow cytometry analysis to monitor the fluorescence intensity of the cells.

#### In vitro kinase assays

Flag‐FHL2 was immunoprecipitated by anti‐Flag antibody from the lysates of Flag‐FHL2‐transfected HEK293T cells. After washed by cell lysis buffer for three times, Flag‐FHL2 was incubated with active c‐Abl protein expressed by baculovirus in Sf21 insect cells (Merck‐Millipore, Billerica, MA, USA,14‐529) in protein kinase buffer with ATP for 30 min at 30 °C. The reaction products were resolved by SDS/PAGE and analyzed by immunoblotting.

## Results

### c‐Abl phosphorylates FHL2 on multiple tyrosine residues

**Fig. 1:**
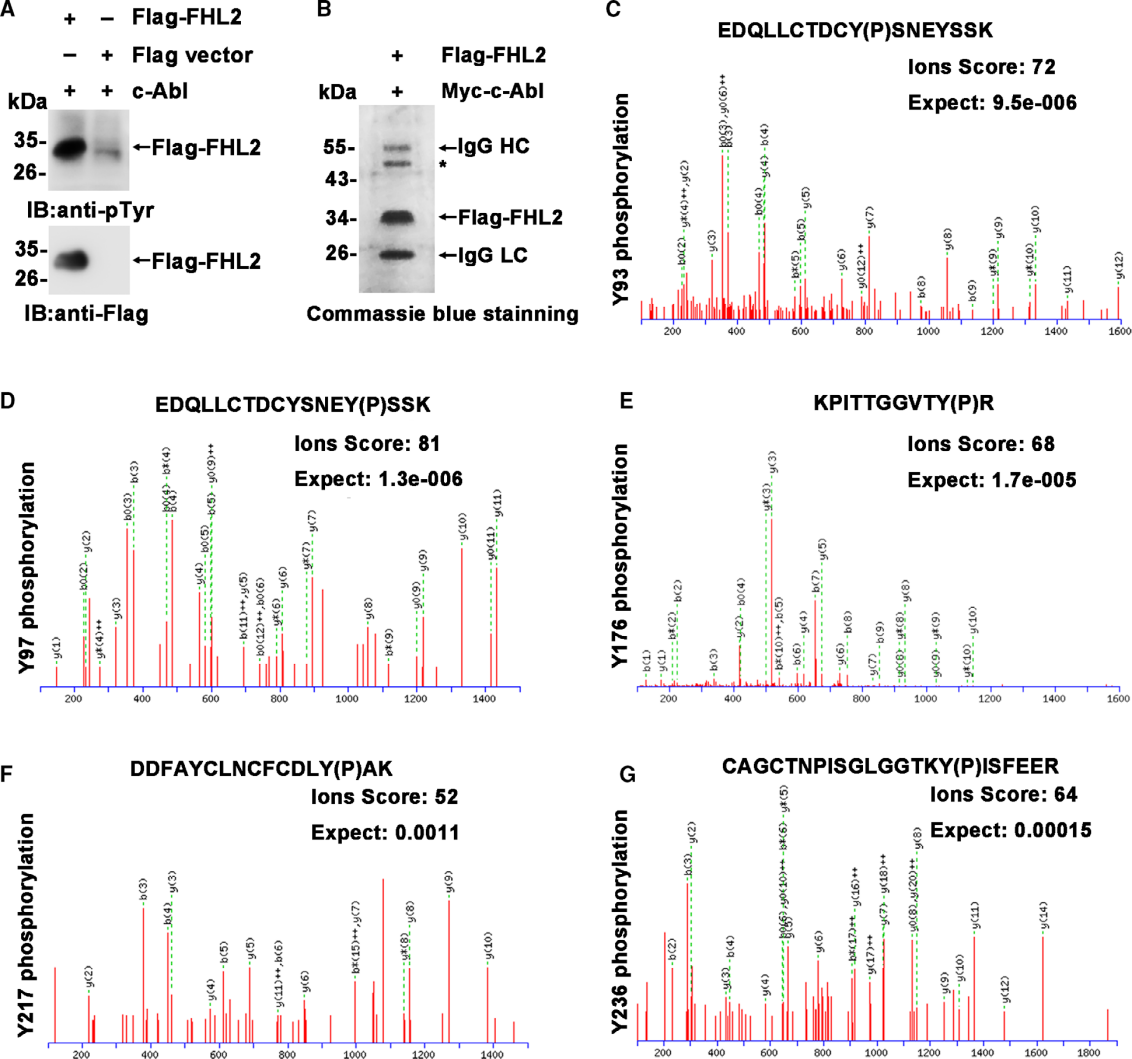
Identification of the phosphorylation sites on FHL2 mediated by c‐Abl. (A) Immunoprecipitates prepared from Flag‐FHL2‐ or Flag vector‐transfected HEK293T cells were incubated with active c‐Abl protein for 30 min at 30 °C. The reaction products were resolved by SDS/PAGE and subjected to immunoblotting. (B) HEK293T cells were cotransfected with Flag‐FHL2 and Myc‐c‐Abl, and the anti‐Flag immunoprecipitates were fractionated by SDS/PAGE. The gel was stained with Coomassie Brilliant Blue (CBB), and the band containing Flag‐FHL2 was excised, trypsin‐digested, and subjected to LC–MS/MS analysis. HC: heavy chain; LC: light chain. *: Unknown protein. C–G. Mass spectra of the peptides with phosphorylated tyrosine residues Y(p). The peptide sequences are shown at the top of the panels, and the ion scores and expect values are shown in the panels.

To demonstrate the direct phosphorylation of FHL2 by c‐Abl, an *in vitro* kinase assay using purified c‐Abl protein was performed (Fig. 1A). To identify the c‐Abl‐dependent phosphorylation site(s) in FHL2, Flag‐FHL2 coexpressed with Myc‐c‐Abl was subjected to immunoprecipitation with anti‐Flag antibody and the immunoprecipitates were then separated by SDS/PAGE. The Flag‐FHL2 protein band was excised from the gel and subjected to trypsin digestion, followed by LC–MS/MS analysis (Fig. 1B). Five potential tyrosine residues (Y93, Y97, Y176, Y217, and Y236) of FHL2 were identified to be phosphorylated by c‐Abl, with an ion score ranging from 52 to 81, where an ion score > 38 was considered ‘significant’ (Fig. 1C‐G).

### FHL2 is phosphorylated by c‐Abl mainly at four tyrosines

**Fig. 2:**
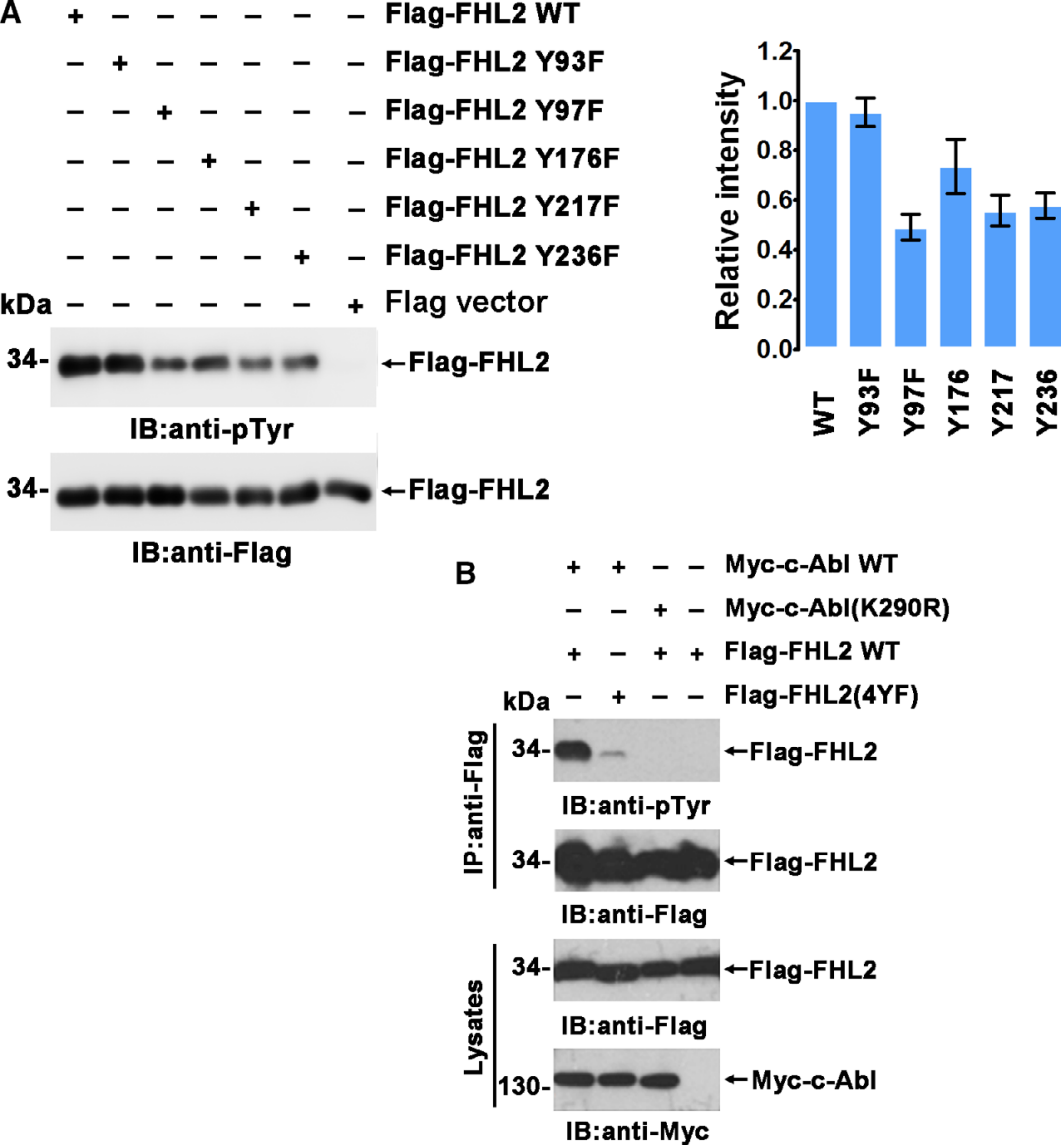
c‐Abl phosphorylates FHL2. (A) HEK293T cells were cotransfected with plasmids expressing Myc‐c‐Abl and Flag‐FHL2 or Flag‐FHL2 mutants. Anti‐Flag immunoprecipitates were analyzed by immunoblotting with anti‐pTyr or anti‐Flag antibody (left). The relative pTyr levels of Flag‐FHL2 and the mutants were quantified and plotted (right). Values are expressed as means ± SD. (B) HEK293T cells were cotransfected with plasmids expressing Myc‐c‐Abl WT/(K290R) and Flag‐FHL2 WT/(4YF). Anti‐Flag immunoprecipitates were analyzed by immunoblotting with anti‐pTyr, anti‐Flag, and anti‐Myc antibodies.

To further confirm the result of mass spectrometry, the five tyrosine residues were mutated from Y to F. The phosphorylation levels of the mutants in the presence of c‐Abl were determined using an anti‐phosphotyrosine (pTyr) antibody. The phosphorylation level was obviously decreased when Y97, Y176, Y217, and Y236 were mutated, but not when Y93F was mutated (Fig. 2A). Moreover, Flag‐FHL2(4YF) that harbors the four Y97/176/217/236F mutations demonstrated nearly abolished phosphorylation compared with wild‐type (WT) FHL2, further confirming that these four sites were the major phosphorylation sites of FHL2. The specificity of c‐Abl‐mediated phosphorylation of FHL2 was further demonstrated by the dominant negative mutant of c‐Abl(K290R), which could not phosphorylate FHL2 by coexpression (Fig. 2B).

### FHL2 tyrosine phosphorylation deficiency results in accelerated cell proliferation

**Fig. 3:**
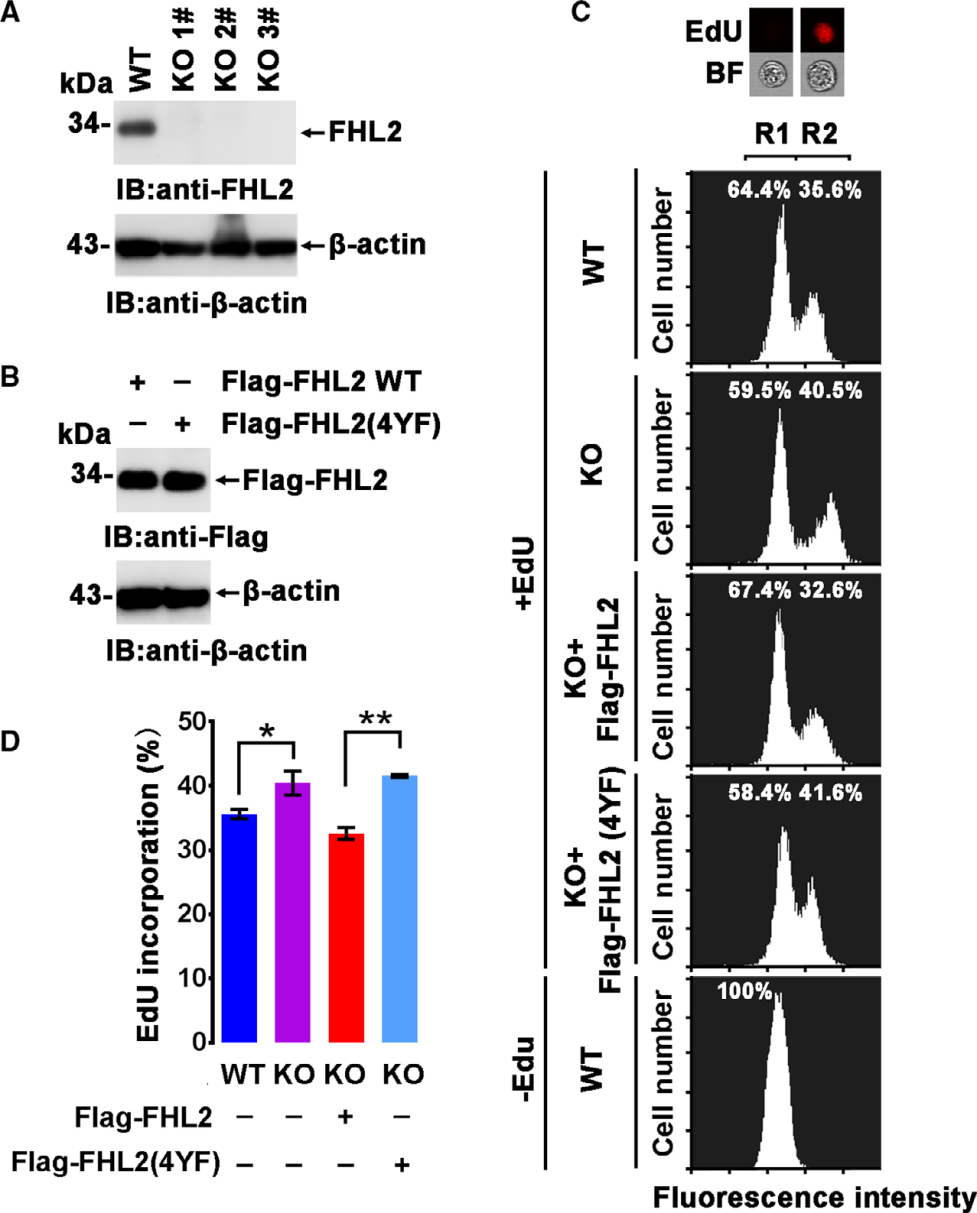
Cell proliferation is regulated by c‐Abl‐mediated phosphorylation on FHL2. (A) FHL2‐knockout MCF‐7 cell clones established using the CRISPR‐Cas9 strategy were confirmed by immunoblotting using an anti‐FHL2 antibody. (B) MCF‐7 KO cells were transfected with the indicated plasmids, and the expression was detected by immunoblotting. (C) MCF‐7 KO cells transfected with the indicated plasmids were incubated with EdU for 12 h or not, and then, the EdU incorporation ratios were analyzed by flow cytometry analysis. The two peaks of each panel indicated the non‐EdU‐incorporated and EdU‐incorporated cells (the R1 peak indicates the non‐EdU‐incorporated cell population; the R2 peak indicates the EdU‐incorporated cell population), and the EdU incorporation ratio is shown at each panel. The representative images of cells with or without EdU incorporation are shown on the top of the graph. BF: bright field. The assay was repeated three times. (D) The mean ratio of EdU incorporation from three independent experiments is shown. Values are expressed as means ± SD. Statistical significance in each case was analyzed using Student’s *t*‐test. *p < 0.05, **p < 0.01.

Because FHL2 is involved in cell cycle regulation, we then investigated whether c‐Abl‐mediated FHL2 phosphorylation impacts FHL2‐involved cell cycle regulation. Thus, an FHL2‐depleted MCF‐7 cell line (FHL2 KO, *fhl2* ^−/−^) was established using the CRISPR‐Cas9 strategy to exclude the influence of endogenous FHL2 (Fig. 3A). Next, the KO cells were transfected with the Flag‐FHL2 WT or Flag‐FHL2(4YF) plasmid with Cas9 resistance synonymous mutations (Fig. 3B), and the EdU incorporation assay was employed to assess cell proliferation. The EdU incorporation ratios increased from 35.5% in WT cells to 40.5% in FHL2 KO cells. Notably, EdU incorporation decreased to a level less than that observed in WT MCF‐7 cells when WT Flag‐FHL2 was ectopically expressed in MCF‐7 KO cells (32.6%). By contrast, Flag‐FHL2(4YF) expression in MCF‐7 KO cells showed little to no effect. These results suggest that FHL2 inhibits cell proliferation dependent on c‐Abl‐mediated tyrosine phosphorylation (Fig. 3C,D).

### FHL2 phosphorylation by c‐Abl promotes DNA damage‐induced G2/M arrest

**Fig. 4:**
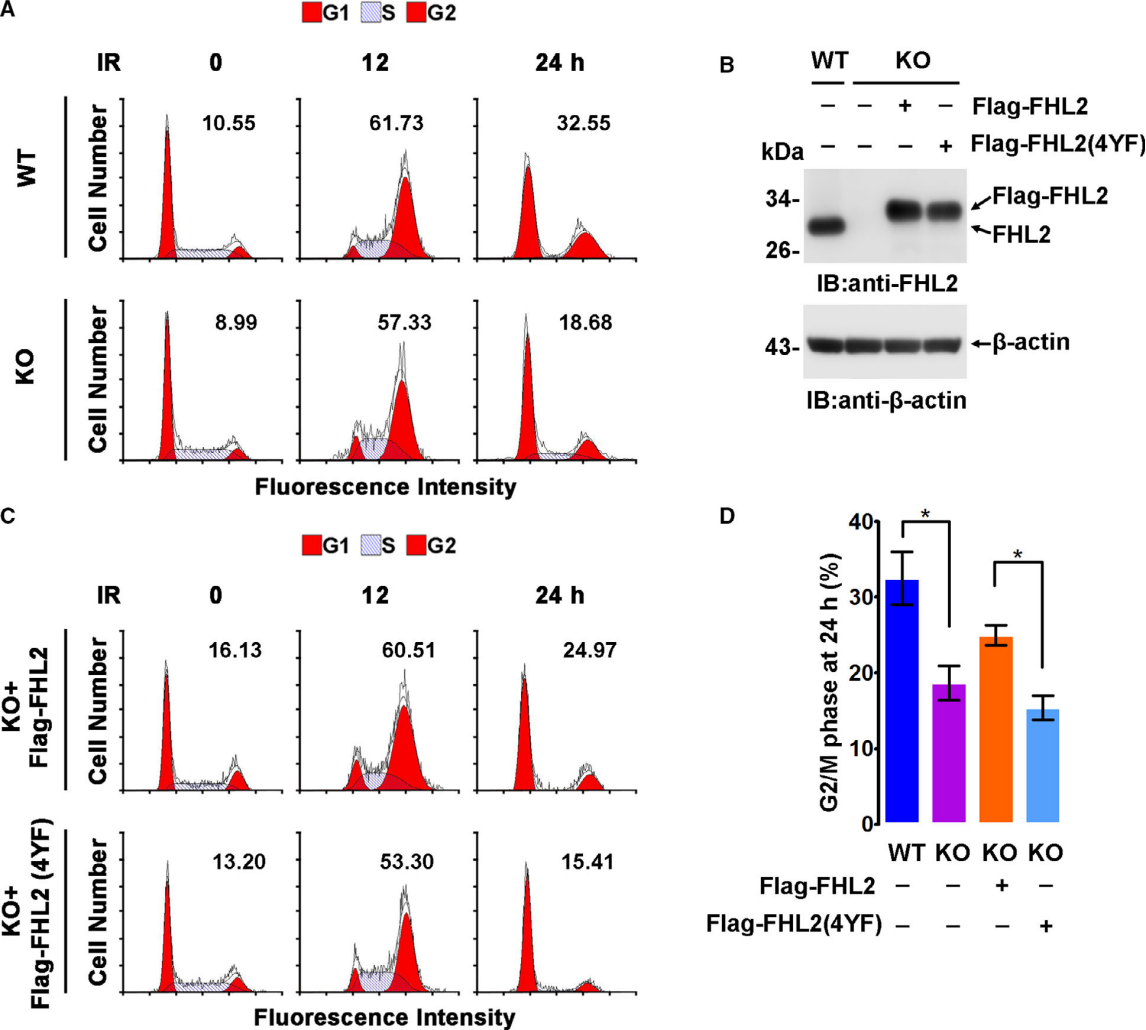
c‐Abl‐mediated FHL2 phosphorylation regulates FHL2‐involved G2/M arrest in response to irradiation. (A) MCF‐7 WT or FHL2 KO cells were treated with γ‐ray at a dosage of 10 Gy, and the cell cycle was analyzed by flow cytometry. The mean value of cell percentage at the G2/M phase in triplicate experiments is shown at the top of each panel. (B) FHL2 in WT MCF‐7 and KO cells transfected with Flag‐FHL2 or Flag‐FHL2(4YF) plasmid or not was detected by immunoblotting. (C) MCF‐7 KO cells were transfected with Flag‐FHL2 or Flag‐FHL2(4YF) plasmid and then were treated and analyzed as described in A. D. The ratios of G2/M phase cells in A and C 24 h after IR are shown as means ± SD. Statistical significance in each case was analyzed using Student’s *t*‐test. *p < 0.05.

FHL2 is involved in G2/M arrest regulation when FHL2 is overexpressed or when cells are treated with DNA damaging agents such as doxorubicin [20, 22]. To explore whether FHL2 phosphorylation impacts G2/M arrest caused by IR, WT and FHL2 KO cells were treated with γ‐irradiation, and the cell cycle was detected at the indicated time intervals. IR‐induced G2/M arrest was observed at 12 h after IR treatment, and a slower release of G2/M was also observed in WT cells than in KO cells at 24 h after IR (Fig. 4A,D). The cell cycle was also detected in KO cells expressing equivalent Flag‐FHL2 WT or Flag‐FHL2(4YF) to endogenous FHL2 in WT cells (Fig. 4B). Consistently, the expression of WT FHL2 in KO cells resulted in prolonged G2/M arrest, while the expression of Flag‐FHL2(4YF) showed little to no effect (Fig. 4C,D). These results suggest that c‐Abl‐mediated FHL2 phosphorylation contributes to FHL2‐involved G2/M arrest in response to irradiation.

## Discussion

FHL2 is an adaptor protein that regulates the transcription of multiple genes. Previous studies on how FHL2 exerts its function have focused on its expression level and protein–protein interactions but rarely on post‐translational modifications. The latter may alter FHL2 interactions with its partners or its subcellular translocation. In a patient with familial dilated cardiomyopathy (DCM), a Gly48Ser mutation that impairs the interactions of FHL2 with the metabolic enzyme titin/connectin may lead to cardiac dysfunction and heart failure [23]. FHL2 was found to be phosphorylated by focal adhesion (FA) kinase on Y93, which promoted the translocation of FHL2 from FA complexes to the nucleus. In the nucleus, FHL2 concentrated at RNA polymerase (Pol) II sites, where it acted as a transcriptional cofactor, promoting *p21* gene expression that inhibits cell proliferation on soft surfaces [24]. In this study, we found that c‐Abl phosphorylated FHL2 on four major tyrosines, except the reported Y93. The phosphorylation by c‐Abl endowed FHL2 with functions in regulating cell proliferation and IR‐induced cell cycle arrest.

We also found that phosphorylated FHL2 antagonizes cell proliferation in MCF‐7 cells, but this phenotype may not be completely consistent in all types of cells because FHL2 functions as either an activator or repressor of target genes in a cell type‐dependent manner [19]. This difference may be due to the different phosphorylation sites or phosphorylation levels on these sites of FHL2 in different cellular processes and in different cell types. FHL2 can promote cell proliferation in some cell types, such as glioblastoma cells, ovarian granulosa cells, and tongue squamous cells, but antagonizes cell proliferation in other cell types, such as breast cancer cells (MCF‐7), colon cancer cells (HT‐29), and smooth muscle cells (SMCs) [22, 25, 26, 27, 28, 29].

In MCF‐7 cells, FHL2 inhibited cell proliferation through repressing the DNA binding protein Id3, but the mechanism was unclear [25]. Phosphorylation might change the interaction between FHL2 and other transcriptional cofactors involved in cell proliferation regulation, such as Id3. However, whether phosphorylation on these sites regulates FHL2‐mediated protein interactions remains to be clarified.

Radiotherapy is an important classical cancer therapy approach, but cancer cells often develop radioresistance to survive IR treatment [30]. Cell cycle check point‐related proteins are involved in radioresistance. c‐Abl plays important roles in IR‐induced DNA damage responses by phosphorylating p53 and p73 [12, 31, 32], and the radiosensitivity of pancreatic cancer cells is increased by FHL2 knockdown, suggesting that FHL2 expression may induce radioresistance [33]. Another FHL family protein, FHL1, has been demonstrated to contribute to radioresistance in cultured HeLa and MCF‐7 cells [34]. In this study, tyrosine‐phosphorylated FHL2 in the cells was involved in the delayed release from G2/M arrest induced by irradiation, prolonging the time for cells to repair damaged DNA and decreasing the sensitivity to irradiation treatment in tumor cells, such as MCF‐7.

c‐Abl and FHL2 are also involved in several other cellular processes, such as cell adhesion, mobility, and cytoarchitecture. However, whether these processes are mediated by c‐Abl‐mediated FHL2 phosphorylation is unclear and warrants further attention.

## Conflict of interest

The authors declare no conflict of interest.

## Author contributions

XL, CC, and GW designed experiments. GW, XN, YB, DW, and HL performed the experiments. GW wrote the paper. QD and HL analyzed the data. XL and CC supervised the research and revised the manuscript.

## Data Availability

The data that support the findings of this study are available from the corresponding author [caoc@nic.bmi.ac.cn] upon reasonable request.
